# Accuracy of minimal residual disease detection by circulating tumor DNA profiling in lung cancer: a meta-analysis

**DOI:** 10.1186/s12916-023-02849-z

**Published:** 2023-05-12

**Authors:** Ran Zhong, Rui Gao, Wenhai Fu, Caichen Li, Zhenyu Huo, Yuewen Gao, Yi Lu, Feng Li, Fan Ge, Hengjia Tu, Zhixuan You, Jianxing He, Wenhua Liang

**Affiliations:** 1 Department of Thoracic Surgery and Oncology, The First Affiliated Hospital of Guangzhou Medical University, Guangzhou, 510120 China; 2 State Key Laboratory of Respiratory Disease, Guangzhou, 510120 China; 3 National Clinical Research Center for Respiratory Disease, Guangzhou, 510120 China; 4 Guangzhou Institute of Respiratory Health, Guangzhou, 510120 China; 5 National Center for Respiratory Medicine, Guangzhou, 510120 China; 6 Nanshan School, Guangzhou Medical University, Guangzhou, 511436 China; 7 First Clinical School, Guangzhou Medical University, Guangzhou, 511436 China

**Keywords:** ctDNA, MRD, Lung cancer, Liquid biopsy, Diagnostic accuracy

## Abstract

**Background:**

The sensitivity and specificity of minimal residual disease detected by circulating tumor DNA profiling (ctDNA MRD) in lung cancer, with particular attention to the distinction between landmark strategy and surveillance strategy, for predicting relapse in lung cancer patients after definitive therapy has yet to be determined.

**Methods:**

The prognostic value of ctDNA MRD by landmark strategy and surveillance strategy was evaluated in a large cohort of patients with lung cancer who received definitive therapy using a systemic literature review and meta-analysis. Recurrence status stratified by ctDNA MRD result (positive or negative) was extracted as the clinical endpoint. We calculated the area under the summary receiver operating characteristic curves, and pooled sensitivities and specificities. Subgroup analyses were conducted based on histological type and stage of lung cancer, types of definitive therapy, and ctDNA MRD detection methods (detection technology and strategy such as tumor-informed or tumor-agnostic).

**Results:**

This systematic review and meta-analysis of 16 unique studies includes 1251 patients with lung cancer treated with definitive therapy. The specificity of ctDNA MRD in predicting recurrence is high (0.86–0.95) with moderate sensitivity (0.41–0.76), whether shortly after treatment or during the surveillance. The landmark strategy appears to be more specific but less sensitive than the surveillance strategy.

**Conclusions:**

Our study suggests that ctDNA MRD is a relatively promising biomarker for relapse prediction among lung cancer patients after definitive therapy, with a high specificity but suboptimal sensitivity, whether in landmark strategy or surveillance strategy. Although surveillance ctDNA MRD analysis decreases specificity compared with the landmark strategy, the decrease is minimal compared to the increase in sensitivity for relapse prediction of lung cancer.

**Supplementary Information:**

The online version contains supplementary material available at 10.1186/s12916-023-02849-z.

## Background

Lung cancer is a commonly diagnosed malignancy that can be categorized into two types based on histological features: non-small-cell lung cancer (NSCLC) comprising approximately 85% of cases, and small-cell lung cancer (SCLC) accounting for around 15% [1]. The widespread utilization of low-dose computed tomography (LDCT) screening has led to an increased identification of lung cancers [2]. Simultaneously, the accelerated advancement of therapeutic interventions for lung cancer has resulted in improved treatment outcomes [3]. More lung cancer patients can potentially attain a cure through treatment modalities, such as surgery, radiotherapy, targeted therapy, immunotherapy, and chemotherapy [4].

Traditional clinical surveillance for lung cancer patients following curative-intent therapies involves serial radiographic imaging, which can only detect macroscopic disease recurrence [5]. In recent years, circulating tumor DNA (ctDNA) minimal residual disease (MRD) has emerged as a promising biomarker for predicting relapse before radiographic evidence is apparent. Identifying MRD provides an opportunity to guide clinical decisions regarding adjuvant or consolidation therapy, potentially leading to earlier intervention in those who may benefit. However, the methods and strategies for detecting ctDNA MRD in patients with lung cancer have varied among different studies, posing challenges for clinicians in interpreting MRD results for patients.

In studies investigating ctDNA MRD in lung cancer, there are two primary types of analyses: landmark analysis and surveillance analysis. Landmark analysis is used to determine the ctDNA status of patients at a single, pre-specified timepoint, usually shortly after the completion of definitive treatment (e.g., surgery and radiotherapy). On the other hand, surveillance analysis involves the evaluation of longitudinal blood draws at multiple time points during follow-up, with ctDNA status determined based on whether any blood draw, regardless of the time point, is positive [6]. Furthermore, with regard to the detection of ctDNA MRD, there are two main strategies: tumor-informed and tumor-agnostic. The tumor-informed approach involves monitoring known tumor-specific variants in post-treatment plasma, while the tumor-agnostic method detects ctDNA using a pre-designed panel that is independent of the primary tumor tissue. The tumor-agnostic strategy is also referred to as the tumor-naïve or uninformed strategy [7, 8].

To investigate the role of ctDNA MRD in landmark and surveillance settings for lung cancer, we conducted a systematic meta-analysis of published studies to determine the overall sensitivity and specificity of ctDNA MRD as a prognostic biomarker. Subgroup analyses were also conducted based on the histological type, stage of lung cancer, types of definitive therapy, and ctDNA MRD detection methods (including the technology and detection strategy, such as tumor-informed or tumor-agnostic approaches).

## Methods

### Design

This systemic review and meta-analysis was exempt from institutional review board approval based on criteria from National Clinical Research Center for Respiratory Disease. The study was conducted in conformity with the Preferred Reporting Items for Systematic Reviews and Meta-analyses (PRISMA) recommendations [9] and was registered (PROSPERO CRD42022324716).

### Literature search strategy

We searched Cochrane, PubMed, EMBASE, and Ovid MEDLINE databases using the following terms without date restriction (last search November 23rd, 2022): lung cancer AND Circulating Tumor DNA/ctDNA AND minimal residual disease/MRD. The appropriate Medical Subject Heading (MeSH) terms were combined in the search builder. Only studies in English were included.

### Selection of studies and patients

Inclusion criteria for studies in our meta-analysis were (1) randomized clinical trials or prospective/retrospective cohort studies that detected ctDNA MRD in patients with lung cancer, regardless of histological types, (2) participants who had received definitive therapy, and (3) studies that reported the ctDNA MRD results of each patient at least once after definitive therapy. Exclusion criteria included (1) poor sample size (≤ 10), (2) non-English language studies or studies that could not be accessed through the databases searched, and (3) studies that did not report the recurrence status of patients.

In our study, ctDNA MRD was analyzed in both landmark and surveillance settings. Landmark ctDNA MRD was collected at a single, pre-specified timepoint, typically shortly after definitive therapy. Surveillance analysis involved evaluating longitudinal blood draws at multiple time points after definitive therapy during follow-up (at least one blood draw beyond 1 month after definitive therapy), with ctDNA status determined by whether any blood draw (irrespective of time point) was positive. The detailed selection criteria for landmark or surveillance strategy in each included study are shown in Additional file 1: Fig. S1. Patients with stage IV lung cancer were excluded from the meta-analysis. Patients with recurrence were defined as having relapsed, regardless of the type of recurrence (local, regional, or distant). The detailed patient selection criteria and data sources for our meta-analysis are shown in Additional file 2: Table S1.

### Data extraction

Four independent investigators (R.G., Y.G., Z.H., R.Z.) extracted data, and any discrepancies were resolved through discussion and consensus. Senior investigators (W.L. and J.H.) reviewed the results. All relevant information regarding the target outcomes was recorded in a Microsoft Excel database. The screening process for original literature included recording the reasons for the inclusion or exclusion of each patient. Basic data collected from each study included the first author, publication year, clinicopathological features, detection details, numbers of ctDNA MRD-positive and -negative patients, and recurrence status. If possible, mortality data were also collected. Sensitivity and specificity of ctDNA MRD were calculated using true positive (TP), false positive (FP), true negative (TN), and false negative (FN) rates, which were stratified by patients’ recurrence status and ctDNA MRD (negative or positive). Sensitivity and specificity results were obtained by calculating the proportion of ctDNA-positive patients among all relapsed patients and the proportion of non-relapsed patients among all ctDNA-negative patients, respectively.

### Risk of bias assessment

The methodological quality of the diagnostic accuracy in this study was evaluated using the revised Quality Assessment of Diagnostic Accuracy Studies (QUADAS-2) criteria [10]. QUADAS-2 consists of four key domains: (1) patient selection; (2) index test; (3) reference standard; (4) flow and timing. Each domain was assessed for the risk of bias, and signaling questions were used to help determine the level of bias, which were classified as “low risk,” “high risk,” or “unclear risk” (Additional file 3: Fig. S2, Additional file 4: Fig. S3).

### Statistical analysis

Diagnostic parameters, including sensitivity, specificity, positive likelihood ratios (PLR), negative likelihood ratios (NLR), and diagnostic odds ratios (DOR) were computed and evaluated using Meta-Disc software version 1.4. PLR was determined as sensitivity divided by (1-specificity), while NLR was computed as (1-sensitivity)/specificity. A PLR > 5.0 and NLR < 0.2 were generally deemed clinically significant. Significant heterogeneity was observed when *P* < 0.05 or *I*^2^ > 50%, and a random-effect model was used. Otherwise, a fixed-effect model was used. Subgroup analyses were performed based on the histological type (NSCLC, SCLC) and stage (stage I ≥ 50%, stage I < 50%) of lung cancer, types of definitive therapy (surgery), and ctDNA MRD detection methods (detection technology, including hybrid capture-based next-generation sequencing (NGS) and amplicon-based NGS; MRD detection strategy, including tumor-informed or tumor-agnostic). To note, we calculated the proportion of patients with stage I lung cancer in each included study, and then divided them into two groups based on the proportion of stage I patients. (Additional file 5: Table S2).

## Results

### Systematic review and characteristics

**Fig. 1:**
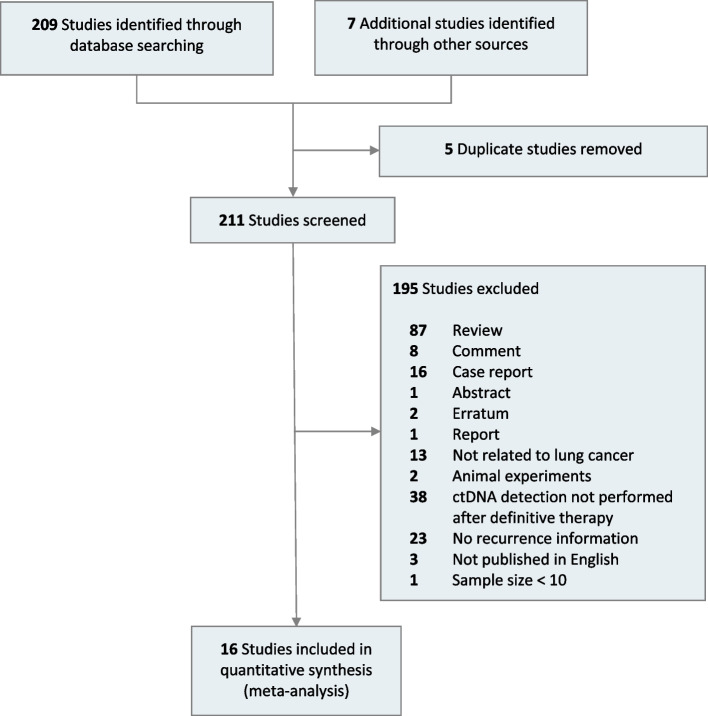
Flow diagram for selection of studies

A total of 209 related studies were identified through database searching, and additional studies were found through the gray literature search. After excluding 5 duplicate studies based on title and abstract, 211 full-text articles were retrieved, and 16 studies were eligible for inclusion (Fig. 1) [7, 8, 11–24]. The analysis included a total of 1251 patients from 16 studies for landmark analysis and 773 patients from 12 studies for surveillance analysis. Patient characteristics for the included studies are presented in Additional file 6: Table S3. Among the included studies, 15 enrolled patients with NSCLC, and 1 focused mainly on SCLC. Regarding the methods and strategies for MRD detection, 11 studies used hybrid capture-based NGS, 4 used amplicon-based NGS, and only 1 used whole genome sequencing (WGS). Of the included studies, 8 used tumor-informed MRD detection, 6 used tumor-agnostic MRD detection, and 2 used a combination of tumor-informed and tumor-agnostic strategies, which were excluded from the subgroup analysis of tumor-informed/agnostic strategy.

### Sensitivity and specificity by landmark strategy

**Fig. 2:**
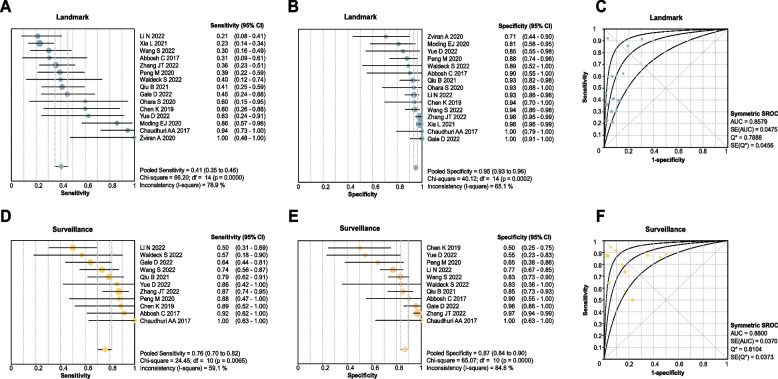
Performance of ctDNA analysis approaches for detecting MRD in non-small cell lung cancer. Size of dots is a visual representation for the weight of that study in the meta-analysis. Error bars, the 95% confidence intervals. **A** Summary of clinical sensitivity for ctDNA detection at the first posttreatment time point (ctDNA MRD landmark). Clinical sensitivity is defined as the percentage of patients who relapsed in the follow-up period and who were ctDNA positive at the landmark. **B** Summary of clinical specificity for ctDNA detection at the first posttreatment time point. Clinical specificity is defined as the percentage of patients who did not relapse in the follow-up period who were ctDNA negative at the landmark. **C** Summary receiver operating characteristic (SROC) curve of ctDNA detection at the first posttreatment time point. **D** Summary of clinical sensitivity for ctDNA detection with longitudinal monitoring posttreatment (ctDNA Surveillance). **E** Summary of clinical specificity for ctDNA detection with longitudinal monitoring posttreatment. **F** SROC curve of ctDNA detection with longitudinal monitoring posttreatment

The analysis of the landmark strategy revealed that the sensitivity of MRD in predicting relapse for NSCLC is 0.41 (95% confidence interval [CI]: 0.35–0.46; Fig. 2A), with a specificity of 0.95 (95% CI: 0.93 to 0.96; Fig. 2B), and an area under curve (AUC) of 0.86 (Fig. 2C). The PLR, NLR, and DOR of the landmark study were 5.56 (95% CI: 3.86–8.01), 0.66 (95% CI: 0.57–0.76), and 11.17 (95% CI: 6.95–17.94), respectively. The sensitivity and specificity of MRD in predicting relapse for lung cancer (SCLC and NSCLC) were similar to the results of NSCLC alone (sensitivity: 0.41, 95% CI: 0.35–0.46; specificity: 0.95, 95% CI: 0.93–0.96) with an AUC of 0.84 (Additional file 7: Fig. S4).

The sensitivity of MRD for relapse prediction was lower in studies with stage I NSCLC patients ≥ 50% (0.31, 95% CI: 0.24–0.38) compared to studies with patients with higher stages of NSCLC (stage I < 50%) (0.51, 95% CI: 0.43–0.59); both groups showed a specificity of over 0.90.

**Fig. 3:**
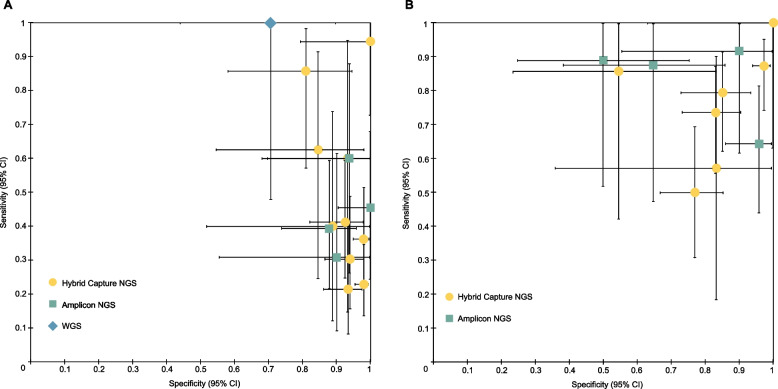
Clinical sensitivity and specificity for ctDNA detection. Error bars, binomial 95% confidence intervals. **A** Subgroup analysis of clinical sensitivity and specificity for ctDNA detection at the first posttreatment time point (ctDNA MRD landmark) for non-small cell lung cancer (NSCLC). **B** Subgroup analysis of clinical sensitivity and specificity for ctDNA detection with longitudinal monitoring posttreatment (ctDNA Surveillance) for NSCLC

**Fig. 4:**
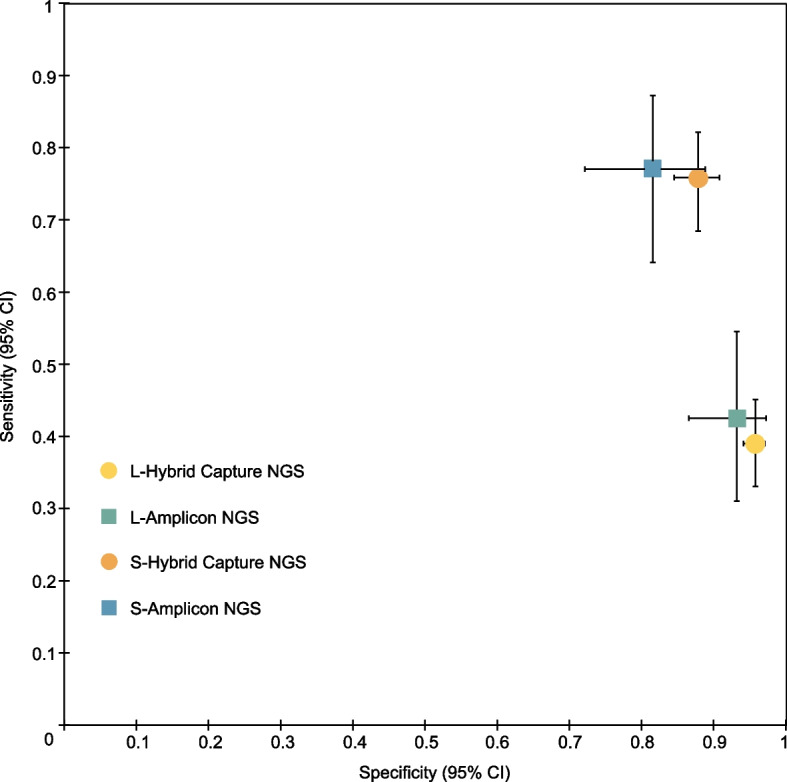
Subgroup analysis of clinical accuracy for different ctDNA detection methods. The ctDNA detection at the first posttreatment time point (ctDNA MRD landmark) or longitudinal monitoring posttreatment (ctDNA surveillance) for non-small cell lung cancer were indicated by different icons of different colors. Error bars, binomial 95% confidence intervals. Each icon represents a summary of the results of studies using a specific detection method under different monitoring methods. L refers to landmark while S refers to surveillance

The diagnosis accuracy of landmark MRD for different ctDNA MRD detection technologies is shown in Figs. 3A and 4. In the subgroup analysis of MRD detection methods using hybrid capture-based NGS, the sensitivity of MRD in predicting relapse for NSCLC is 0.39 (95% CI: 0.33–0.45), and the specificity was 0.96 (95% CI: 0.94–0.97). In amplicon-based NGS, the sensitivity of MRD in predicting relapse of NSCLC is 0.42 (95% CI: 0.31–0.55), and the specificity was 0.93 (95% CI: 0.87–0.97).

In the landmark strategy, among patients who underwent surgery as definitive therapy, the sensitivity of MRD in predicting relapse for NSCLC is 0.35 (95% CI: 0.29–0.40; Additional file 8: Fig. S5A), and the overall specificity was 0.95 (95% CI: 0.94–0.97; Additional file 8: Fig. S5B), with an AUC of 0.77 (Additional file 8: Fig. S5C). In the landmark strategy of NSCLC, tumor-informed ctDNA MRD showed a sensitivity of 0.32 (95% CI:0.26–0.39) and a specificity of 0.95 (95% CI:0.93–0.97), with an AUC of 0.78. In the landmark strategy of NSCLC, the tumor-agnostic method had a sensitivity of 0.61 (95% CI:0.48–0.72) and a specificity of 0.91 (95% CI:0.84–0.96), with an AUC of 0.97 (Table 1).

**Table 1:** Results of the meta-analysis for landmark and surveillance MRD in different groups

Subgroup analysis		Landmark							Surveillance						
		TP	FP	FN	TN	Sensitivity	Specificity	AUC	TP	FP	FN	TN	Sensitivity	Specificity	AUC
Lung cancer type^b^	Lung cancer (stage I–III)	145	47	213	846	0.41 (0.35–0.46)	0.95 (0.93–0.96)	0.84	178	73	55	467	0.76 (0.70–0.82)	0.86 (0.83–0.89)	0.88
	NSCLC (stage I–III)	140	44	205	836	0.41 (0.35–0.46)	0.95 (0.93–0.96)	0.86	169	70	53	460	0.76 (0.70–0.82)	0.87 (0.84–0.90)	0.88
	SCLC^a^	3	3	8	9	0.27 (0.06–0.61)	0.75 (0.43–0.95)		9	3	2	6	0.82 (0.48–0.98)	0.67 (0.30–0.93)	
Stage	Patients at stage I ≥ 50% (NSCLC)	55	25	123	581	0.31 (0.24–0.38)	0.96 (0.94–0.97)	0.81	62	31	21	262	0.75 (0.64–0.84)	0.89 (0.85–0.93)	0.87
	Patients at stage I < 50% (NSCLC)	85	19	82	255	0.51 (0.43–0.59)	0.93 (0.89–0.96)	0.90	107	39	32	198	0.77 (0.69–0.84)	0.84 (0.78–0.88)	0.87
	Patients at stage I < 50% (lung cancer)	90	22	90	265	0.50 (0.42–0.58)	0.92 (0.89–0.95)	0.88	116	42	34	207	0.77 (0.70–0.84)	0.83 (0.78–0.87)	0.87
Definitive therapy	Surgery (NSCLC)	108	40	197	801	0.35 (0.29–0.40)	0.95 (0.94–0.97)	0.77	153	70	48	449	0.76 (0.70–0.82)	0.87 (0.83–0.89)	0.87
Detection method	Amplicon NGS (NSCLC)	31	7	42	97	0.42 (0.31–0.55)	0.93 (0.87–0.97)	0.43	44	17	13	75	0.77 (0.64–0.87)	0.82 (0.72–0.89)	0.90
	Hybrid Capture NGS (NSCLC)	104	32	163	727	0.39 (0.33–0.45)	0.96 (0.94–0.97)	0.88	125	53	40	385	0.76 (0.68–0.82)	0.88 (0.84–0.91)	0.87
	Hybrid Capture NGS (lung cancer)	109	35	171	737	0.39 (0.33–0.45)	0.95 (0.94–0.97)	0.85	134	56	42	392	0.76 (0.69–0.82)	0.88 (0.84–0.90)	0.86
Detection strategy	Tumor-informed (NSCLC)	69	27	146	533	0.32 (0.26–0.39)	0.95 (0.93–0.97)	0.78	99	46	44	243	0.69 (0.61–0.77)	0.84 (0.79–0.88)	0.87
	Tumor-agnostic (NSCLC)	42	9	27	92	0.61 (0.48–0.72)	0.91 (0.84–0.96)	0.97	29	19	3	33	0.91 (0.75–0.98)	0.63 (0.49–0.76)	0.97
	Tumor-agnostic (lung cancer)	47	12	35	102	0.57 (0.46–0.68)	0.89 (0.82–0.94)	0.98	38	22	5	40	0.88 (0.75–0.96)	0.65 (0.51–0.76)	0.94

*Abbreviations*: *TP* true positive, *FP* false positive, *FN* false negative, *TN* true negative, *AUC* area under curve, *NA*, not applicable

^a^Only one SCLC study (stage I–III, stage I ≥ 50%, tumor-agnostic). ^b^After using 2 h after surgery as preferred landmark in Peng M’s study, the landmark results changed to: lung cancer (stage I–III): sensitivity: 0.41 (0.36–0.46), specificity: 0.95 (0.93–0.96), AUC: 0.84; NSCLC (stage I–III): sensitivity: 0.41 (0.36–0.47), specificity: 0.95 (0.93–0.96), AUC: 0.86

### Sensitivity and specificity by surveillance strategy

For NSCLC, the receiver operating characteristic (ROC) analysis of surveillance MRD revealed an AUC of 0.88 (Fig. 2F), with sensitivity and specificity of 0.76 (95% CI: 0.70–0.82; Fig. 2D) and 0.87 (95% CI: 0.84–0.90; Fig. 2E), respectively. The PLR of surveillance study is 4.67 (95% CI: 2.54–8.57), the NLR is 0.29 (95% CI: 0.19–0.45), and the DOR is 21.14 (95% CI: 8.06–55.43). The sensitivity and specificity for lung cancer (SCLC and NSCLC) were also numerically similar to the NSCLC MRD in surveillance analysis, with an AUC of 0.88 (Additional file 4: Fig. S3). In surveillance analysis of NSCLC, ctDNA MRD showed an AUC of 0.87 regardless of studies enrolled patients with stage I < 50% or ≥ 50% (Table 1).

The hybrid capture-based NGS MRD using surveillance strategy demonstrated a sensitivity of 0.76 (95% CI: 0.68–0.82) and a specificity of 0.88 (95% CI: 0.84–0.91) in predicting relapse of NSCLC, with an AUC of 0.87. The amplicon-based NGS MRD demonstrated a sensitivity of 0.77 (95% CI: 0.64–0.87) and a specificity of 0.82 (95% CI: 0.72–0.89) in predicting relapse of NSCLC, with an AUC of 0.90. Among patients treated with surgery, the sensitivity of MRD in predicting relapse for NSCLC is 0.76 (95% CI: 0.70–0.82; Additional file 7: Fig. S5D), and the overall specificity is 0.87 (95% CI: 0.83–0.89; Additional file 7: Fig. S5E), with an AUC of 0.87 (Additional file 7: Fig. S5F) in surveillance analysis. In the surveillance strategy for NSCLC, the tumor-informed MRD showed an AUC of 0.87, and the tumor-agnostic method showed an AUC of 0.97 (Table 1).

Based on our meta-analysis, the landmark strategy appears to be more specific but less sensitive than the surveillance strategy in predicting relapse in lung cancer patients after definitive therapy. However, while surveillance ctDNA MRD analysis may reduce specificity compared to the landmark strategy, the decrease is minimal when compared to the increase in sensitivity for predicting relapse of lung cancer. Table 1 shows the detailed results of our meta-analysis.

### Heterogeneity and inconsistency assessment

Funnel plots were used to assess the heterogeneity of pairwise meta-analyses (Additional file 9: Fig. S6). To investigate whether different histological types would affect the results, we performed separate meta-analyses after excluding an SCLC study, and found similar results (Table 1). To further evaluate the impact of the preferred landmark time point (2 h post-surgery) in the study by Peng M et al. [12], we conducted an additional meta-analysis by using data at 2 h post-surgery (Additional file 5: Table S2), which revealed similar results (Table 1).

## Discussion

Previous evidence indicated that lung cancer patients with positive ctDNA MRD have a worse prognosis than those with negative ctDNA MRD results. However, the current ctDNA MRD method and strategy vary in different lung cancer studies; the difference between landmark strategy and surveillance strategy for predicting relapse in lung cancer patients after definitive therapy remained undetermined. We reviewed the up-to-date evidence that detection of ctDNA MRD following definitive therapy in predicting relapse of lung cancer, and synthesized the published data to compare the performance of landmark MRD with the surveillance MRD.

Our study suggests that the overall performance of ctDNA MRD for relapse prediction in lung cancer is relatively reliable, with a high specificity whether in landmark or surveillance strategy. The high specificity of ctDNA MRD was consistent with the results from previous studies, suggesting that patients with positive ctDNA MRD are associated with worse outcomes [6]. The difference between landmark analysis and surveillance analysis in our study is whether the included patients had multiple time points of MRD. For surveillance strategy, an increase in sensitivity comes at the cost of a higher false-positive rate. However, the rise in sensitivity is greater than the decrease in specificity.

Although the surveillance strategy appears to improve the sensitivity of ctDNA MRD for relapse prediction in lung cancer, the overall sensitivity from our meta-analysis is still suboptimal, whether in landmark or surveillance strategy. Based on our results, the overall sensitivity of ctDNA MRD still needs to be improved. ctDNA refers to the fraction of cell-free DNA in a patient’s blood that originates from a tumor [25]. Previous studies have reported great technical and analytical challenges in detecting ctDNA as MRD [25, 26]. Different approaches, such as optimizing cfDNA recovery, minimizing the effects of technical and biological background, and tracking multiple tumor genotype-informed mutations, can be employed to improve ctDNA detection [6, 27, 28]. Our meta-analysis showed that the highest pooled sensitivity was achieved in tumor-agnostic MRD detection for NSCLC by surveillance strategy (Table 1). Among the 16 included studies, Zviran et al. presented the highest sensitivity (Only landmark MRD), indicating that genome-wide cell-free DNA mutational integration enables ultra-sensitive cancer monitoring. The other 15 studies used amplicon-based and/or hybrid capture-based NGS for ctDNA MRD detection. Although WGS showed the highest sensitivity among all the included studies in ctDNA MRD for relapse prediction (Fig. 3A), it provides limited confidence in the sensitivity to detect any individual site (for example, a driver mutational event) [24]. The common NGS-based techniques have been tailored for targeted sequencing of specific gene panels and can be subdivided into amplicon or hybrid capture-based sequencing [25, 29, 30]. Our results showed that, in the surveillance strategy, the AUC of amplicon-based NGS was numerically similar to that of the hybrid capture-based NGS. From our findings, the AUC of hybrid-capture-based NGS was numerically lower than that of amplicon hybrid capture-based NGS in landmark strategy. Our study also conducted subgroup analysis based on a tumor-informed/agnostic strategy. The tumor-agnostic strategy seems better than the tumor-informed strategy when we compared their AUC. This might be explained by ultra-high-depth sequencing used in the tumor-agnostic method, which increases genome coverage of sequencing and the sensitivity of mutation detection. However, such an increase in sequencing depth and gene coverage also increases the cost. A head-to-head comparison of the above ctDNA MRD detection technologies/strategies is needed to identify the optimal method. ctDNA MRD analysis may have a role in identifying targetable genomic alterations to guide adjuvant target therapy. In scenarios where therapeutic targeting requires such information, deep-targeted approaches are more appropriate. In terms of the tumor-informed strategy, a recent study indicated that clonal mutations in ctDNA were more prognostically informative than subclonal ones [23].

In addition to focusing on ctDNA, emerging technologies could be integrated into the current MRD methods. DNA methylation or other epigenetic features reflecting the chromatin state of tumor cells have been used to detect ctDNA [31]. A study using this assay to detect MRD demonstrated that incorporating epigenomic analysis enhanced sensitivity compared to tumor-agnostic somatic alterations alone. Another study indicated that combining exoRNA and ctDNA increased the sensitivity for EGFR mutation detection in plasma [32]. A 14-gene expression assay has been used to predict survival in resected NSCLC and guide the adjuvant therapy [33–35]. Perioperative dynamic breathomics might be an approach for identifying lung cancer breath biomarkers [36–38]. Further studies are needed to investigate the underlying mechanisms of these potential methods and incorporate them into the current MRD scenario.

Besides improving the sensitivity of ctDNA MRD from a technological perspective, the following unmet clinical needs for MRD deserve attention. It remains unclear whether ctDNA MRD is associated with the site of recurrence. The sensitivity of MRD monitoring was found to be limited in patients with brain-only recurrence [21]. The non-shedder group should be identified before using ctDNA MRD. Although our study indicated a high specificity of ctDNA MRD detection for lung cancer, the relatively low sensitivity should not be overlooked; caution is needed when interpreting these results, especially for those with negative results.

Overall, this is an up-to-date meta-analysis that comprehensively compared the performance of landmark and surveillance strategies in ctDNA MRD for relapse prediction among patients with lung cancer after definitive therapy. Our findings suggest that the surveillance strategy in ctDNA MRD could enhance enrichment in patients at high risk of recurrence. However, current approaches to detect MRD in lung cancer still lack the sensitivity required for clinical application.

### Limitations

Although our study provides a comprehensive and up-to-date evaluation of ctDNA MRD for relapse prediction in lung cancer by landmark and surveillance strategy, it faces several limitations. First, our meta-analysis was based on 16 studies, 8 of which had a relatively small sample size (less than 50). Second, lung cancer is a heterogeneous tumor; variables such as histological type, stage, driver gene status, and adjuvant treatment mode can affect the prognosis of patients. However, most of the included studies did not present such detailed information for individuals. Our study conducted subgroup analyses based on the classification in histological (SCLC and NSCLC), stage (proportion of patients with stage I lung cancer: ≥ 50% or < 50%), and definitive therapy (surgery). In this meta-analysis, we were unable to capture the impact of driver gene status and adjuvant treatment mode of the enrolled patients on our MRD results, which could affect the prognosis of patients with lung cancer after definitive therapy.

## Conclusions

ctDNA MRD was indicated to be a relatively promising biomarker for relapse prediction among lung cancer patients after definitive therapy, with a high specificity and a suboptimal sensitivity, whether in landmark strategy or surveillance strategy. Although surveillance ctDNA MRD analysis decreases specificity compared with the landmark strategy, the decrease is minimal compared to the increase in sensitivity for relapse prediction of lung cancer. Novel sensitive and cost-effective methods of MRD are urgently needed, which could potentially have a huge impact on risk stratification, treatment, and outcome for patients with lung cancer in the future.

## Supplementary Information

**Additional file 1: Figure S1.** ctDNA MRD detection time points of each included study. Landmark ctDNA MRD is collected at a single, pre-specified timepoint, typically shortly after definitive therapy. Surveillance analysis evaluates longitudinal blood draws at multiple time points after definitive therapy during follow-up (at least one blood draw beyond 1 month after definitive therapy).

**Additional file 2: Table S1.** Description of Landmark and Surveillance strategy of each included study. NA: Not applicable. *: The preferred time point as Landmark.

**Additional file 3: Figure S2.** Methodological quality graph. The following criteria were evaluated: patient selection, index test, reference standard and following time. All four criteria were used for the assessment of risk of bias, and the first three were also used for the assessment of applicability concerns.

**Additional file 4: Figure S3.** Methodological quality summary. Details of the risk of bias and applicability concerns of each study are presented.

**Additional file 5: Table S2.** Information of each included study for meta-analysis between landmark and surveillance strategy. Abbreviations: TP, true positive; FP, false positive; FN, false negative; TN, true negative. *: After excluding patients with small-cell lung cancer, the data for Chaudhuri AA 2017 changed to: landmark: TP = 17, TN = 0, FN = 1, TN = 16; surveillance: TP = 8, TN = 0, FN = 0, TN = 8. ψ: Patients who have been reported in Chaudhuri AA 2017 were excluded. ξ: After using 2 h after surgery as preferred landmark in Peng M 's study, the landmark results changed to: TP = 13, FP = 5, FN = 15, TN = 36.

**Additional file 6: Table S3.** Baseline characteristics of patients in included studies.

**Additional file 7: Figure S4.** Performance of ctDNA analysis approaches for detecting MRD in lung cancer. Size of dots is a visual representation for the weight of that study in the meta-analysis. Error bars, the 95% confidence intervals. A, Summary of clinical sensitivity for ctDNA detection at the first posttreatment time point (ctDNA MRD landmark). Clinical sensitivity is defined as the percentage of patients who relapsed in the follow-up period and who were ctDNA positive at the landmark. B, Summary of clinical specificity for ctDNA detection at the first posttreatment time point. Clinical specificity is defined as the percentage of patients who did not relapse in the follow-up period who were ctDNA negative at the landmark. C, Summary Receiver Operating Characteristic (SROC) curve of ctDNA detection at the first posttreatment time point. D, Summary of clinical sensitivity for ctDNA detection with longitudinal monitoring posttreatment (ctDNA surveillance). E, Summary of clinical specificity for ctDNA detection with longitudinal monitoring posttreatment. F, SROC curve of ctDNA detection with longitudinal monitoring posttreatment.

**Additional file 8: Figure S5.** Performance of ctDNA analysis approaches for detecting MRD in patients after surgery. Size of dots is a visual representation for the weight of that study in the meta-analysis. Error bars, the 95% confidence intervals. A, Summary of clinical sensitivity for ctDNA detection at the first posttreatment time point (ctDNA MRD landmark). Clinical sensitivity is defined as the percentage of patients who relapsed in the follow-up period and who were ctDNA positive at the landmark. B, Summary of clinical specificity for ctDNA detection at the first posttreatment time point. Clinical specificity is defined as the percentage of patients who did not relapse in the follow-up period who were ctDNA negative at the landmark. C, Summary Receiver Operating Characteristic (SROC) curve of ctDNA detection at the first posttreatment time point. D, Summary of clinical sensitivity for ctDNA detection with longitudinal monitoring posttreatment (ctDNA surveillance). E, Summary of clinical specificity for ctDNA detection with longitudinal monitoring posttreatment. F, SROC curve of ctDNA detection with longitudinal monitoring posttreatment.

**Additional file 9: Figure S6.** Funnel plots based on the diagnostic odds ratios (DOR). The dashed lines indicate the funnel. The circles represent individual studies. A, Funnel plot of circulating tumor DNA (ctDNA) detection at the first posttreatment time point (ctDNA MRD landmark). B, Funnel plot of ctDNA detection with longitudinal monitoring posttreatment (ctDNA Surveillance).

## Data Availability

All data generated or analyzed during this study are presented in this published article.

## Abbreviations

ctDNA: Circulating tumor DNA
MRD: Minimal residual disease
NSCLC: Non-small cell lung cancer
SCLC: Small-cell lung cancer
