# Supplementary figures and images for: Accuracy of minimal residual disease detection by circulating tumor DNA profiling in lung cancer: a meta-analysis
Source: BMC Med. 2023 May 12;21:180. doi: 10.1186/s12916-023-02849-z (PMC10176776; doi:10.1186/s12916-023-02849-z)

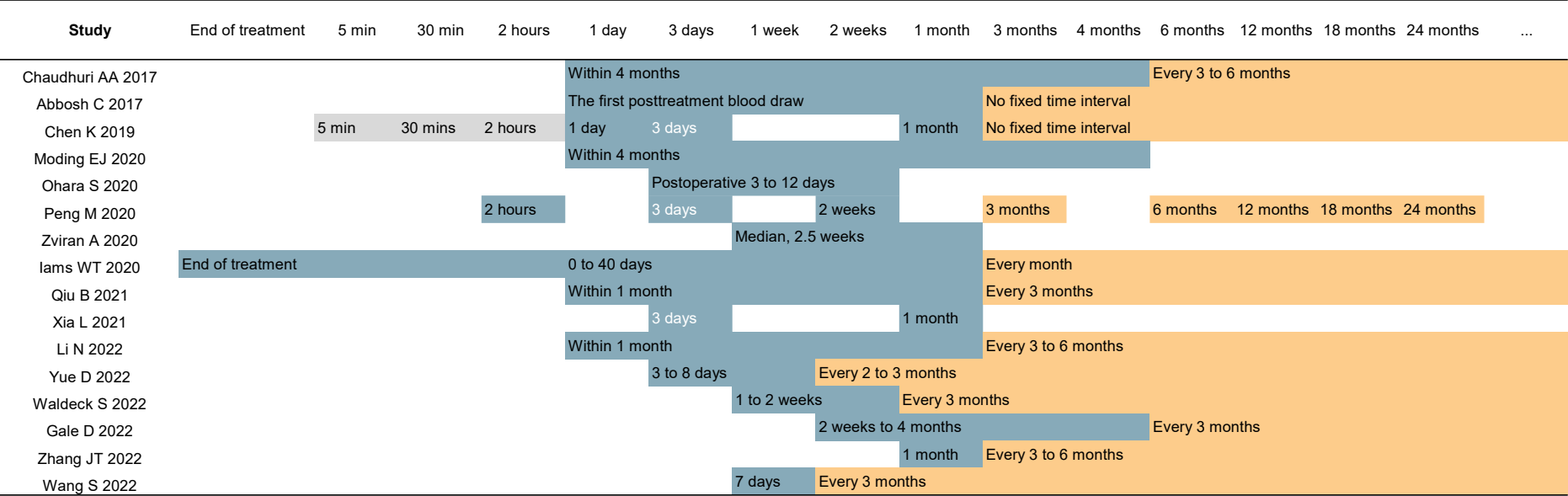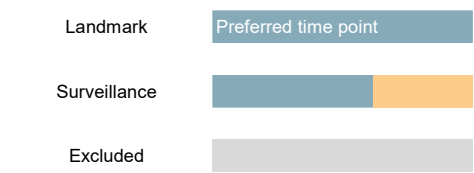

Supplement: Supplementary file 1 — Additional file 1: Figure S1. ctDNA MRD detection time points of each included study. Landmark ctDNA MRD is collected at a single, pre-specified timepoint, typically shortly after definitive therapy. Surveillance analysis evaluates longitudinal blood draws at multiple time points after definitive therapy during follow-up (at least one blood draw beyond 1 month after definitive therapy). [file 12916_2023_2849_MOESM1_ESM.pdf]

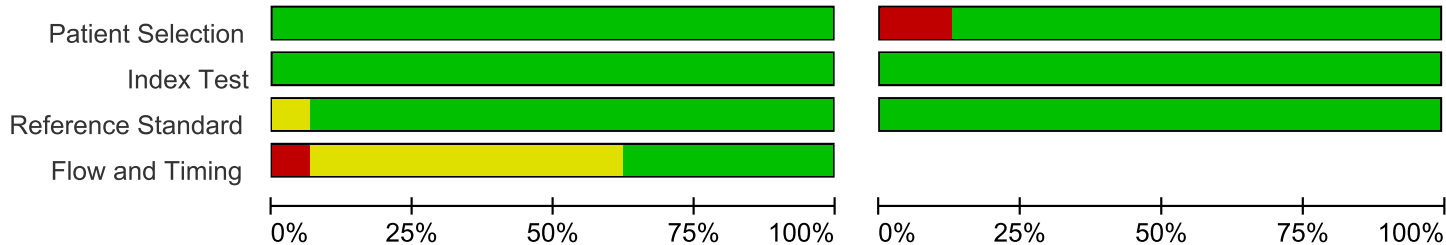

**Risk of Bias**

**Applicability Concerns**

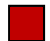

High

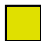

Unclear

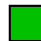

Low

Supplement: Supplementary file 3 — Additional file 3: Figure S2. Methodological quality graph. The following criteria were evaluated: patient selection, index test, reference standard and following time. All four criteria were used for the assessment of risk of bias, and the first three were also used for the assessment of applicability concerns. [file 12916_2023_2849_MOESM3_ESM.pdf]

A

## Landmark

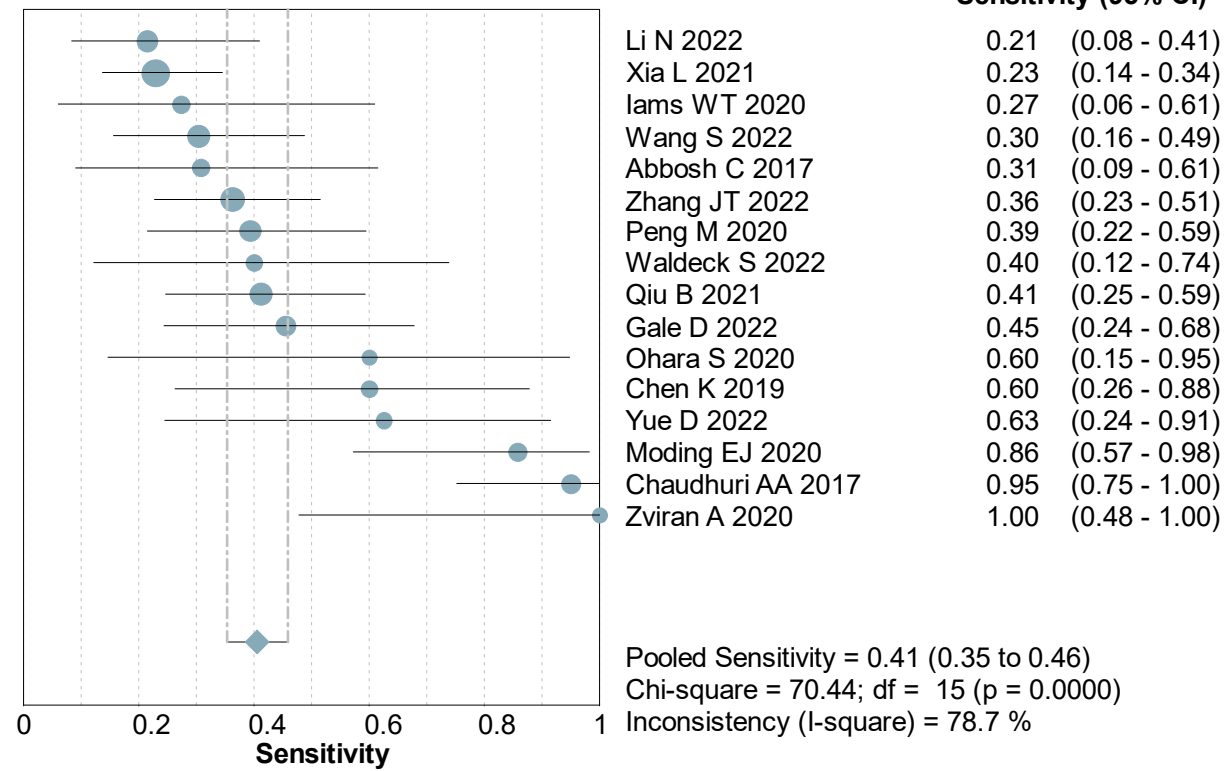

B

## Landmark

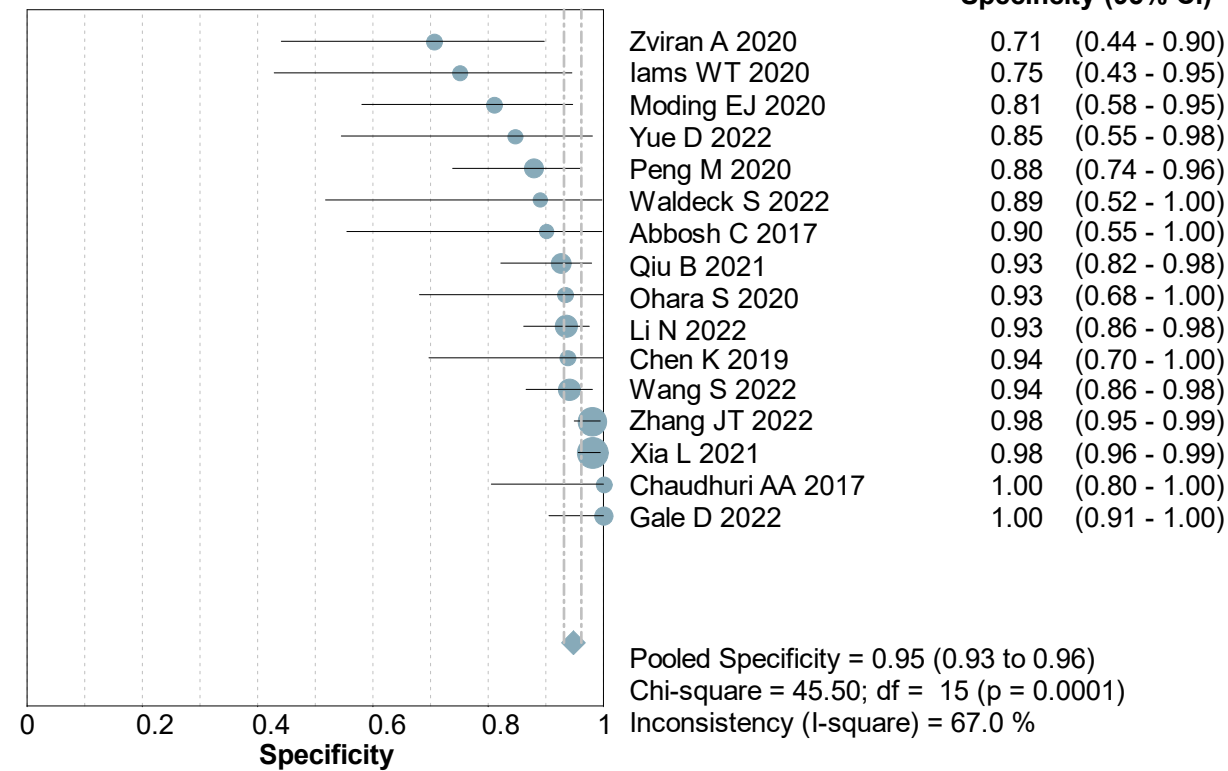

C

## Landmark

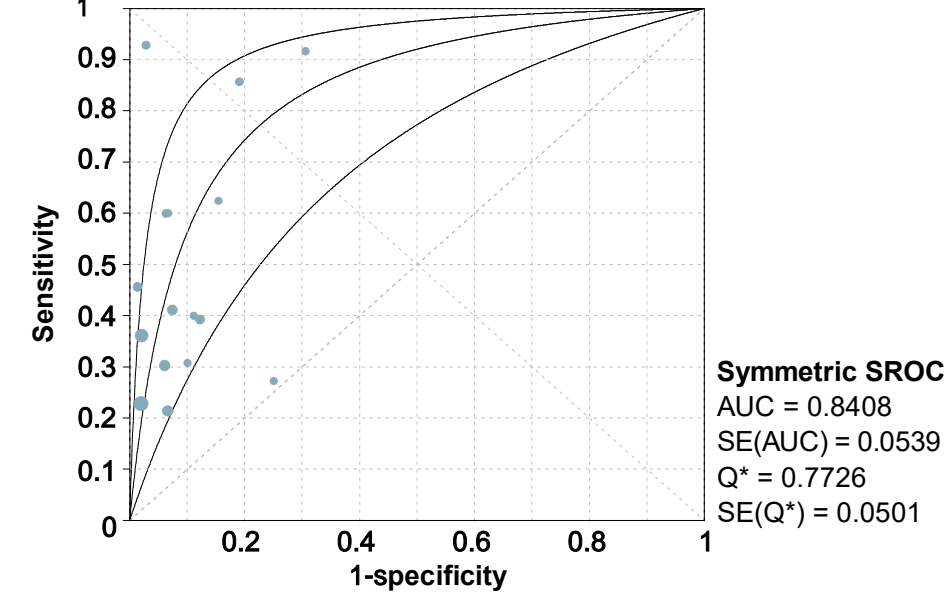

D

## Surveillance

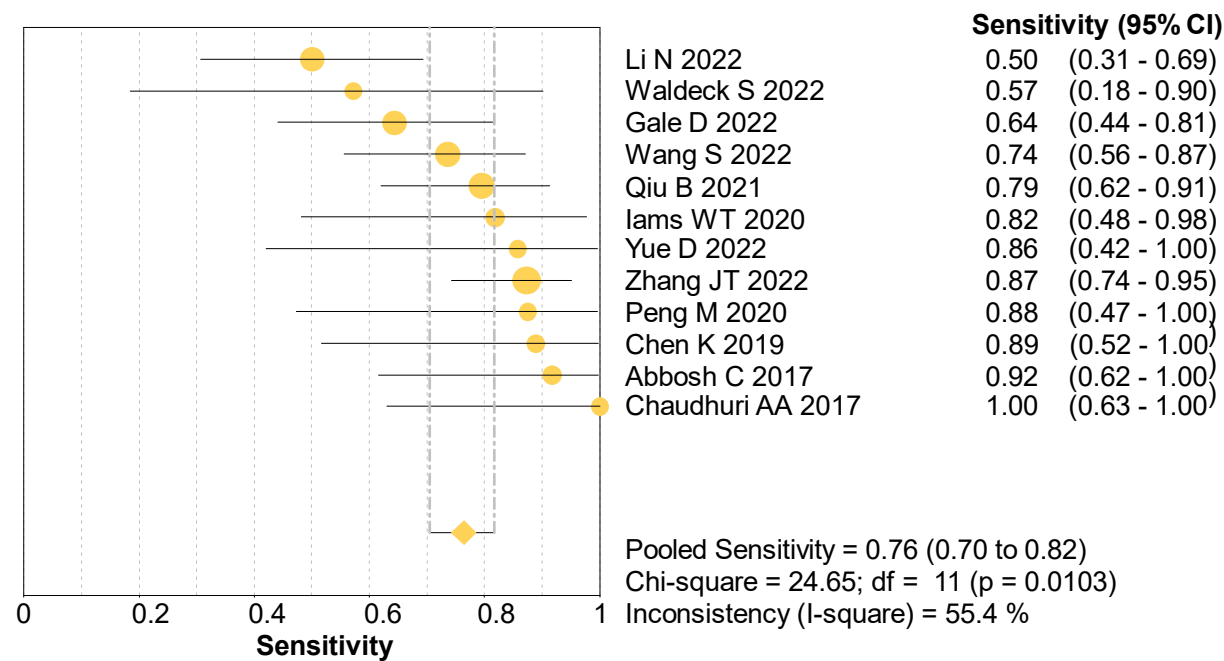

E

## Surveillance

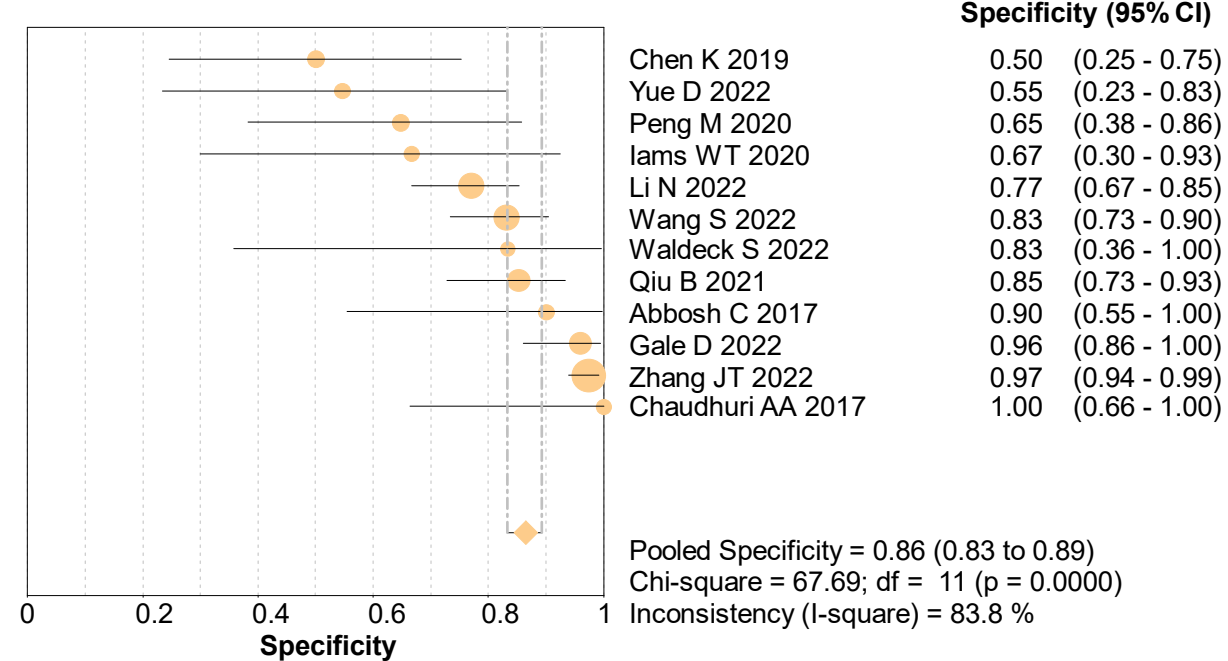

F

## Surveillance

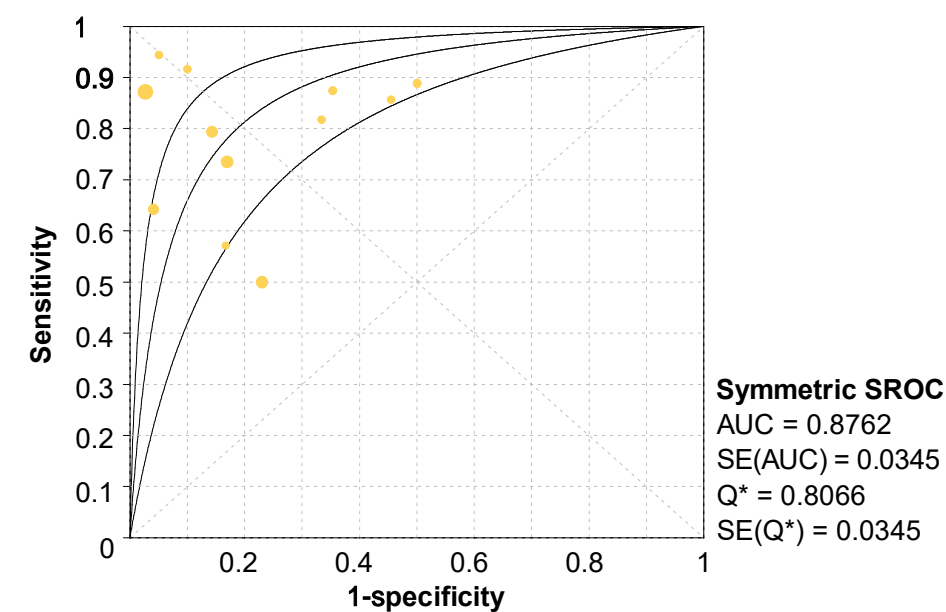

Supplement: Supplementary file 7 — Additional file 7: Figure S4. Performance of ctDNA analysis approaches for detecting MRD in lung cancer. Size of dots is a visual representation for the weight of that study in the meta-analysis. Error bars, the 95% confidence intervals. A, Summary of clinical sensitivity for ctDNA detection at the first posttreatment time point (ctDNA MRD landmark). Clinical sensitivity is defined as the percentage of patients who relapsed in the follow-up period and who were ctDNA positive at the landmark. B, Summary of clinical specificity for ctDNA detection at the first posttreatment time point. Clinical specificity is defined as the percentage of patients who did not relapse in the follow-up period who were ctDNA negative at the landmark. C, Summary Receiver Operating Characteristic (SROC) curve of ctDNA detection at the first posttreatment time point. D, Summary of clinical sensitivity for ctDNA detection with longitudinal monitoring posttreatment (ctDNA surveillance). E, Summary of clinical specificity for ctDNA detection with longitudinal monitoring posttreatment. F, SROC curve of ctDNA detection with longitudinal monitoring posttreatment. [file 12916_2023_2849_MOESM7_ESM.pdf]

A

## Landmark

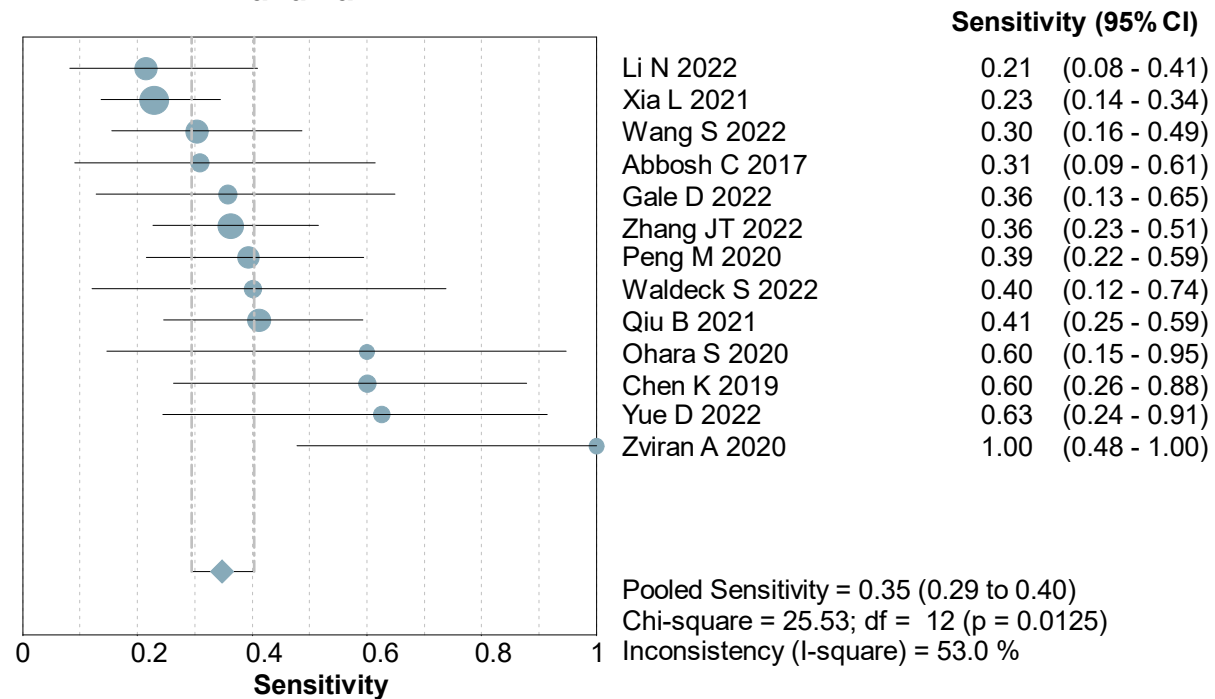

B

## Landmark

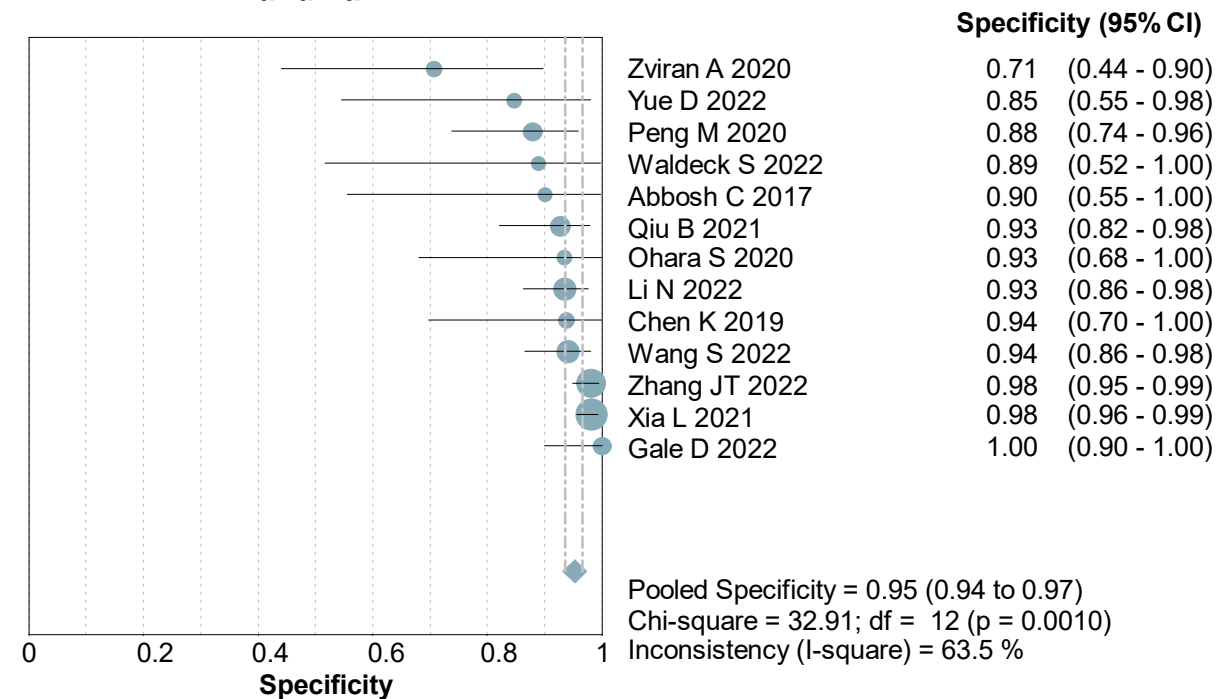

C

## Landmark

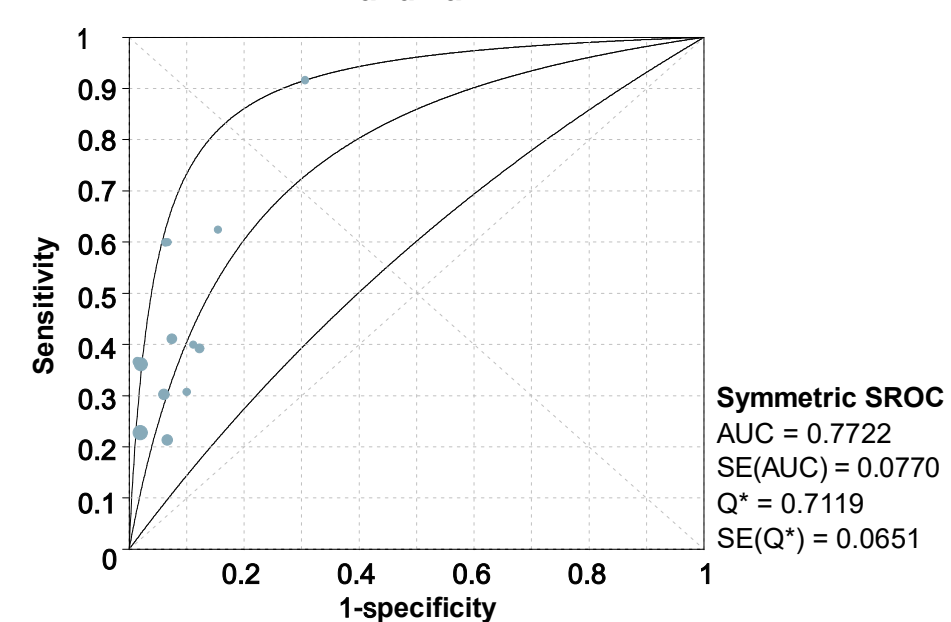

D

## Surveillance

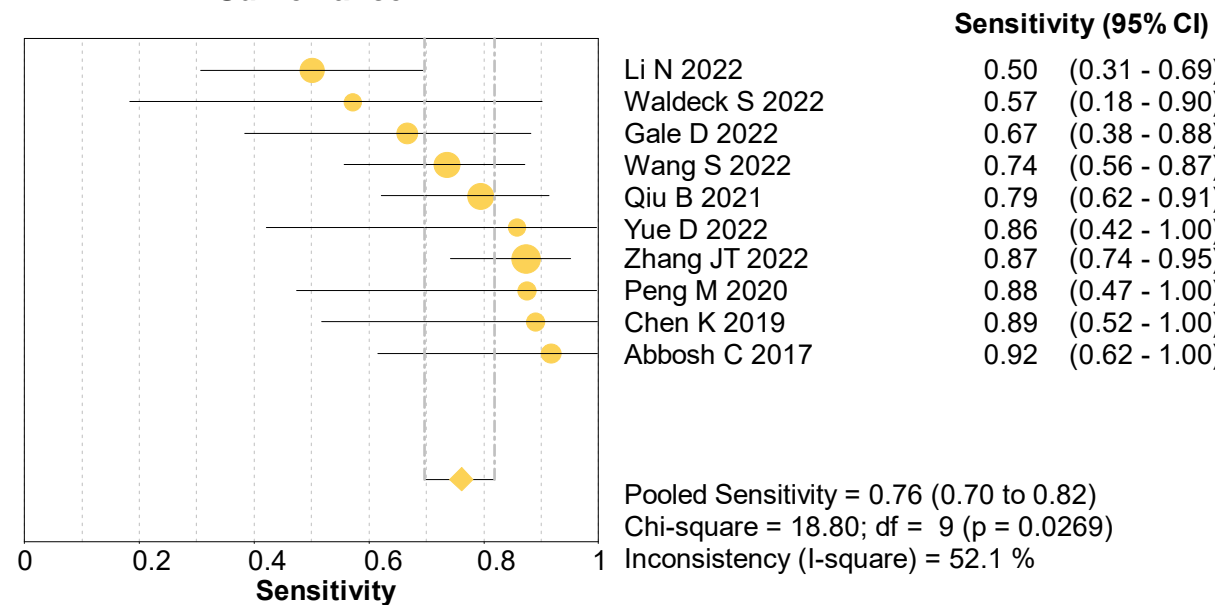

E

## Surveillance

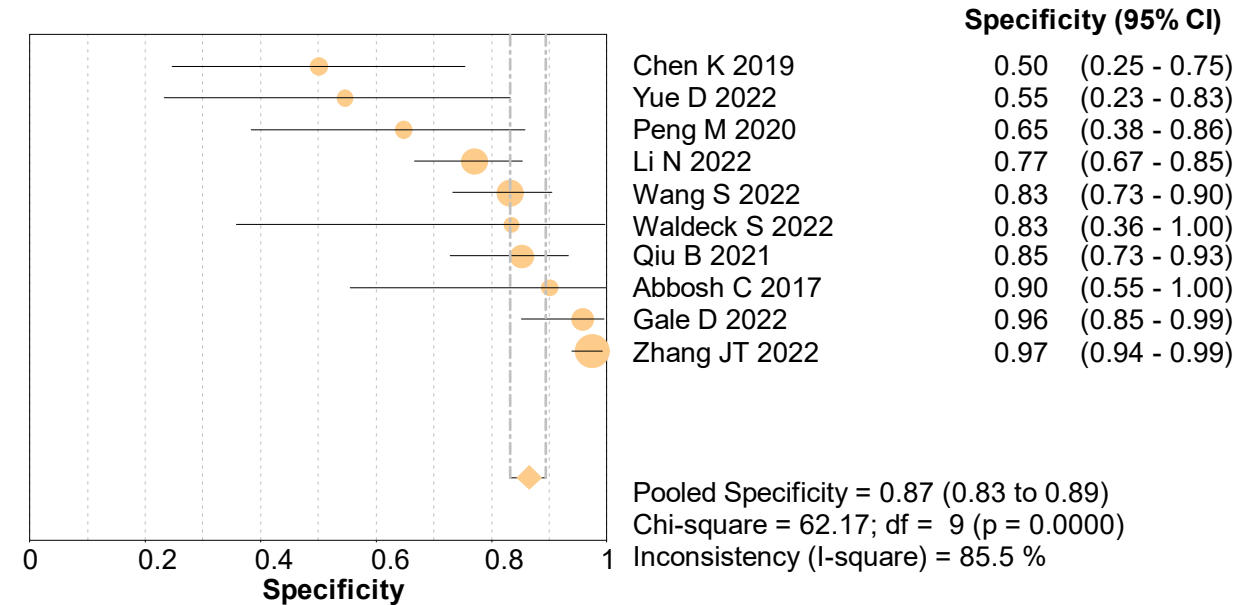

F

## Surveillance

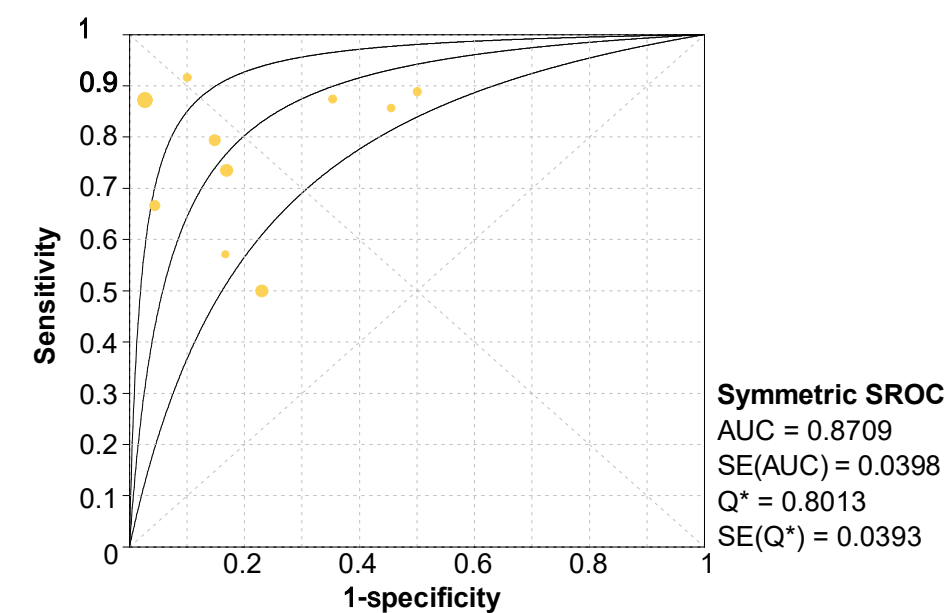

Supplement: Supplementary file 8 — Additional file 8: Figure S5. Performance of ctDNA analysis approaches for detecting MRD in patients after surgery. Size of dots is a visual representation for the weight of that study in the meta-analysis. Error bars, the 95% confidence intervals. A, Summary of clinical sensitivity for ctDNA detection at the first posttreatment time point (ctDNA MRD landmark). Clinical sensitivity is defined as the percentage of patients who relapsed in the follow-up period and who were ctDNA positive at the landmark. B, Summary of clinical specificity for ctDNA detection at the first posttreatment time point. Clinical specificity is defined as the percentage of patients who did not relapse in the follow-up period who were ctDNA negative at the landmark. C, Summary Receiver Operating Characteristic (SROC) curve of ctDNA detection at the first posttreatment time point. D, Summary of clinical sensitivity for ctDNA detection with longitudinal monitoring posttreatment (ctDNA surveillance). E, Summary of clinical specificity for ctDNA detection with longitudinal monitoring posttreatment. F, SROC curve of ctDNA detection with longitudinal monitoring posttreatment. [file 12916_2023_2849_MOESM8_ESM.pdf]

A

Funnel Plot

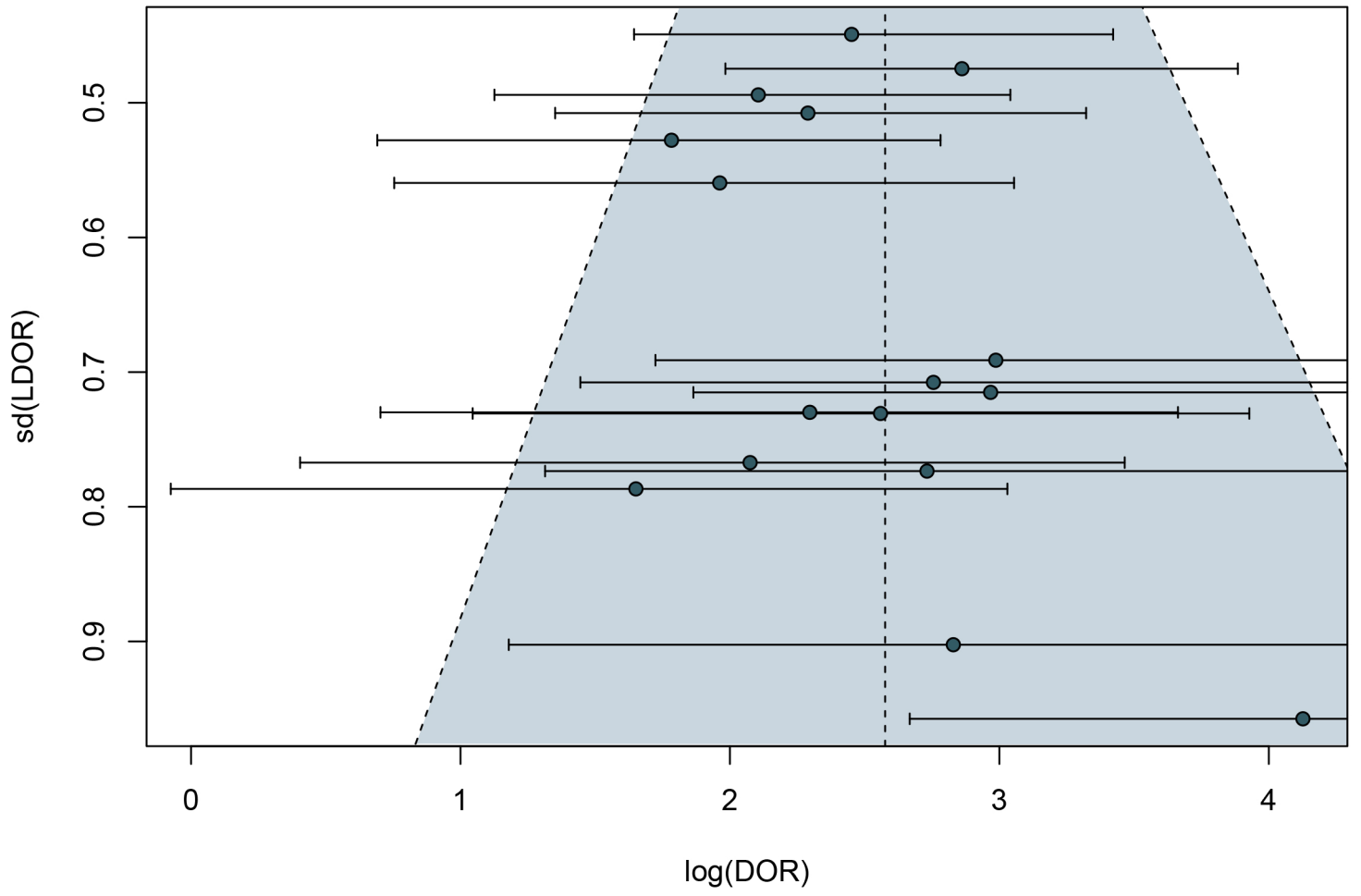

B

Funnel Plot

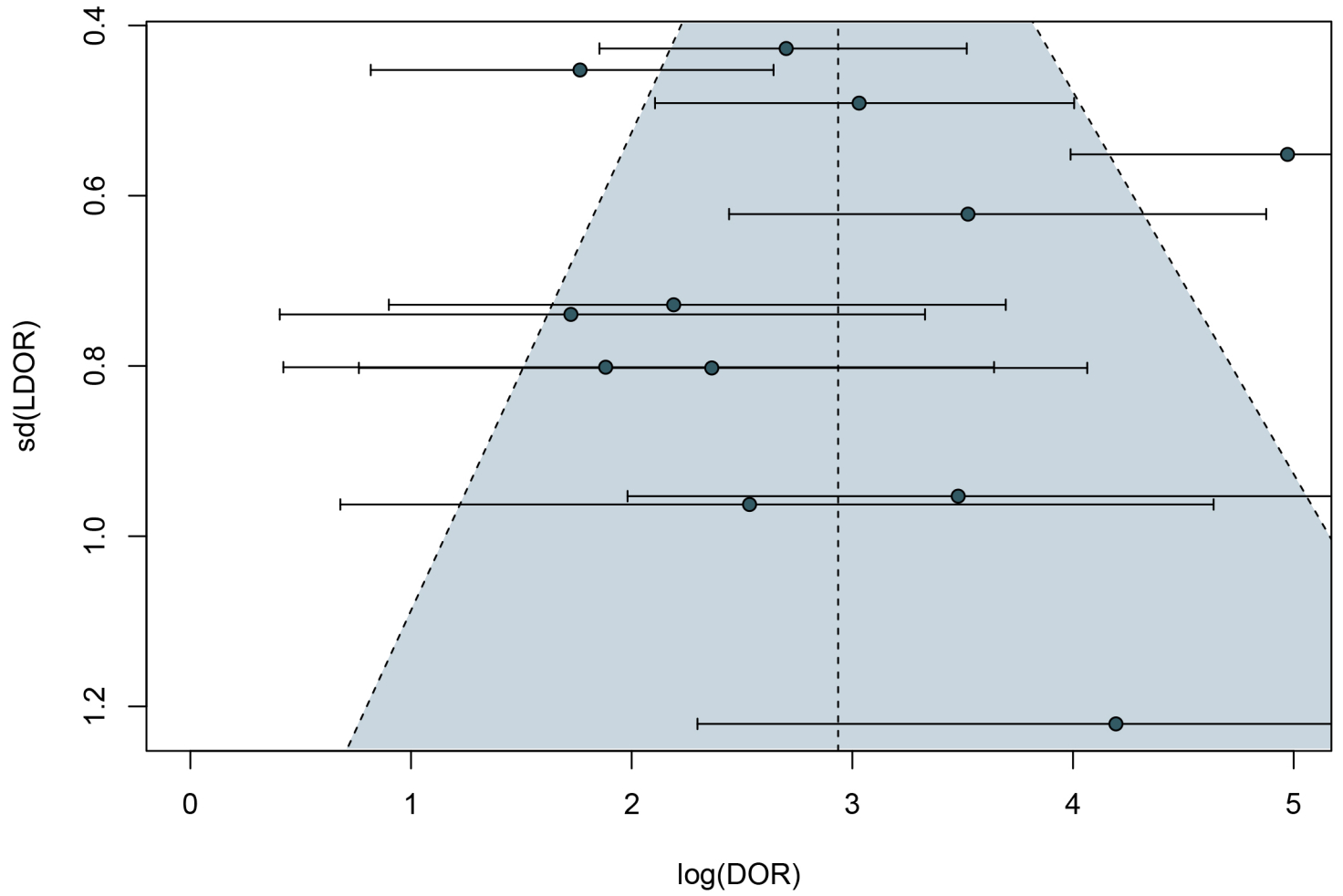

Supplement: Supplementary file 9 — Additional file 9: Figure S6. Funnel plots based on the diagnostic odds ratios (DOR). The dashed lines indicate the funnel. The circles represent individual studies. A, Funnel plot of circulating tumor DNA (ctDNA) detection at the first posttreatment time point (ctDNA MRD landmark). B, Funnel plot of ctDNA detection with longitudinal monitoring posttreatment (ctDNA Surveillance). [file 12916_2023_2849_MOESM9_ESM.pdf]
