# Supplementary material for: Accuracy of minimal residual disease detection by circulating tumor DNA profiling in lung cancer: a meta-analysis
Source: BMC Med. 2023 May 12;21:180. doi: 10.1186/s12916-023-02849-z (PMC10176776; doi:10.1186/s12916-023-02849-z)
Supplement: Supplementary file 4 — Additional file 4: Figure S3. Methodological quality summary. Details of the risk of bias and applicability concerns of each study are presented. [file 12916_2023_2849_MOESM4_ESM.pdf]

	Risk of Bias				Applicability Concerns		
	Patient Selection	Index Test	Reference Standard	Flow and Timing	Patient Selection	Index Test	Reference Standard
Abbosh C 2017	+	+	+	?	+	+	+
Chaudhuri AA 2017	+	+	+	+	+	+	+
Chen K 2019	+	+	+	?	+	+	+
Gale D 2022	+	+	+	?	+	+	+
Iams WT 2020	+	+	+	+	−	+	+
Li N 2022	+	+	+	?	+	+	+
Moding EJ 2020	+	+	+	?	+	+	+
Ohara S 2020	+	+	+	?	+	+	+
Peng M 2020	+	+	+	+	+	+	+
Qiu B 2021	+	+	+	+	+	+	+
Waldeck S 2022	+	+	+	+	+	+	+
Wang S 2022	+	+	+	?	+	+	+
Xia L 2021	+	+	+	?	−	+	+
Yue D 2022	+	+	+	−	+	+	+
Zhang JT 2022	+	+	+	?	+	+	+
Zviran A 2020	+	+	?	+	+	+	+

− **High** ? **Unclear** + **Low**
